# The Specific Vulnerabilities of Cancer Cells to the Cold Atmospheric Plasma-Stimulated Solutions

**DOI:** 10.1038/s41598-017-04770-x

**Published:** 2017-06-30

**Authors:** Dayun Yan, Haitao Cui, Wei Zhu, Niki Nourmohammadi, Julian Milberg, Lijie G. Zhang, Jonathan H. Sherman, Michael Keidar

**Affiliations:** 1 Department of Mechanical and Aerospace Engineering, The George Washington University, Science & Engineering Hall, 800 22nd Street, NW, Room 3550, Washington, DC 20052 USA; 2 Department of Epidemiology, Columbia University, Mailman School of Public Health, 722 West 168th Street, New York, NY 10032 USA; 3 Department of Biomedical Engineering, University of Miami, 1251 Memorial Drive McArthur Engineering Building, Coral Gables, FL 33146-0621 USA; 4 Neurological Surgery, The George Washington University, Foggy Bottom South Pavilion, 22nd Street, NW, 7th Floor, Washington, DC 20037 USA

## Abstract

Cold atmospheric plasma (CAP), a novel promising anti-cancer modality, has shown its selective anti-cancer capacity on dozens of cancer cell lines *in vitro* and on subcutaneous xenograft tumors in mice. Over the past five years, the CAP-stimulated solutions (PSS) have also shown their selective anti-cancer effect over different cancers *in vitro* and *in vivo*. The solutions used to make PSS include several bio-adaptable solutions, mainly cell culture medium and simple buffered solutions. Both the CAP-stimulated medium (PSM) and the CAP-stimulated buffered solution (PSB) are able to significantly kill cancer cells *in vitro*. In this study, we systematically compared the anti-cancer effect of PSM and PSB over pancreatic adenocarcinoma cells and glioblastoma cells. We demonstrated that pancreatic cancer cells and glioblastoma cells were specifically vulnerable to PSM and PSB, respectively. The specific response such as the rise of intracellular reactive oxygen species of two cancer cell lines to the H_2_O_2_-containing environments might result in the specific vulnerabilities to PSM and PSB. In addition, we demonstrated a basic guideline that the toxicity of PSS on cancer cells could be significantly modulated through controlling the dilutability of solution.

## Introduction

Cold atmospheric plasma (CAP) is a near room temperature ionized gas composed of charged particles, neutral particles, and electrons^1^. Among these particles, the oxygen-based reactive species and the nitrogen-based reactive species may mainly contribute to the interaction between CAP and cells^2–6^. Over the past decade, CAP has shown its remarkably selective anti-cancer capacity *in vitro* ^5–9^. Moreover, CAP is able to significantly reduce the size of subcutaneous xenograft tumors in mice models by a direct treatment just above the skin^10–15^. The direct CAP treatments rely upon a CAP generating device during the treatment. Over the past five years, the indirect CAP treatment became a new topic in plasma medicine. The indirect CAP treatment uses the CAP-treated biologically adaptable solutions (PSS) to affect the growth of cancer cells *in vitro* ^13, 16–30^ or *in vivo* by injecting PSS into the tumor tissues^17, 30^. PSS can be stored over a long time length at −80 °C^20, 26^ by optimizing the composition of the solution^24^. Thus, PSS can be used without the dependence on the CAP generating device. In addition, cancer cells were actually covered by a thin layer of medium or other simple buffered solutions during the direct CAP treatment^15, 31, 32^. This thin layer of solution facilitates the transition of the CAP-originated reactive species in the gas phase to be the dissolved species in the aqueous solutions^5, 33^. Thus, the study of PSS will also facilitate the understanding on the direct CAP treatment.

**Figure 1:**
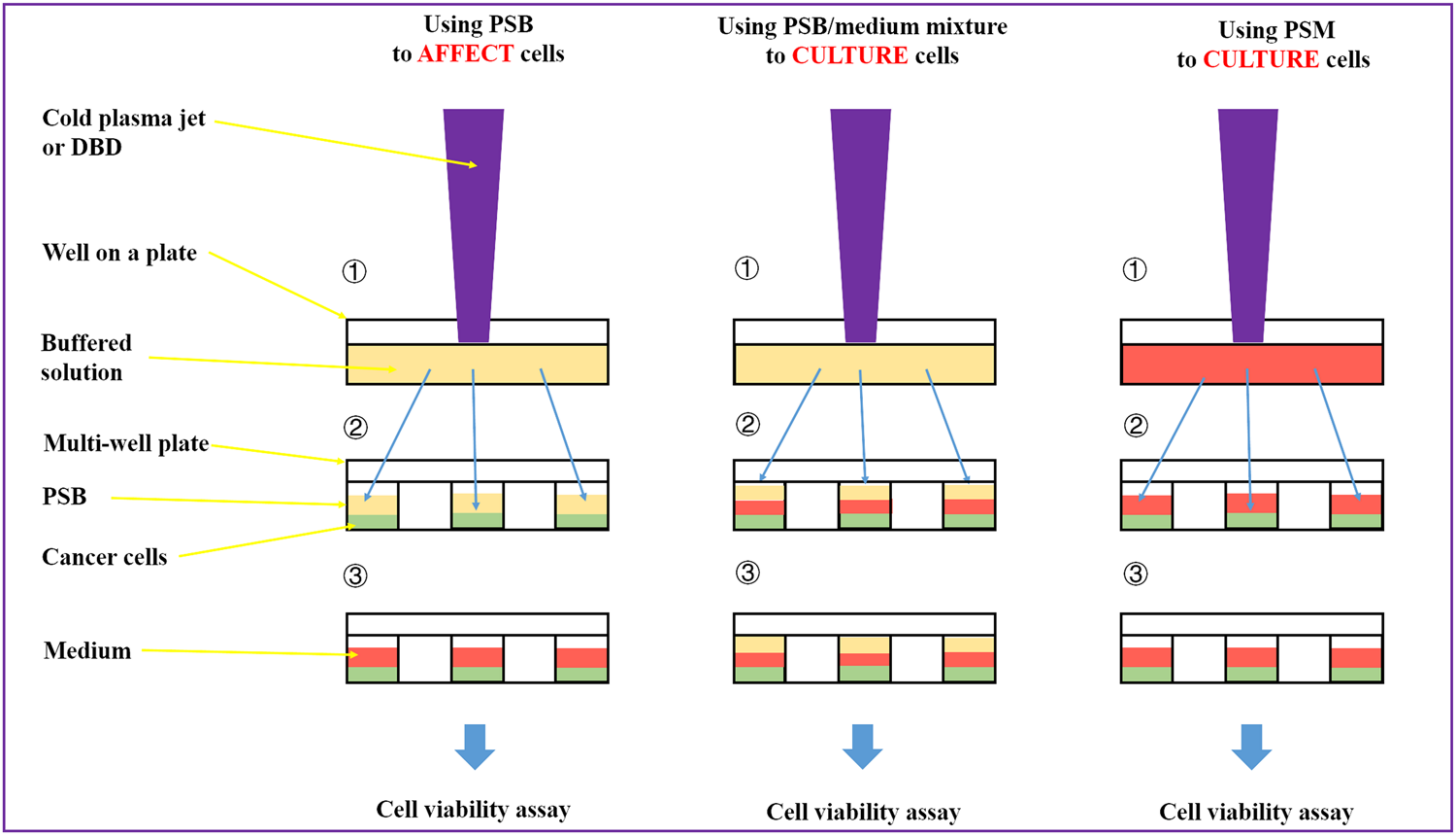
Typical strategies of using PSS to affect the growth of cancer cells *in vitro*. The differences between these strategies are just due to one fact that a simple buffered solution such as PBS and Lactated Ringer’s solution cannot be used to culture cells over a relative long time length. Step ①: Making PSM/PSB by treating the solutions with CAP. Step ②: Using PSM/PSB to affect the growth of cancer cells. Step ③: Culturing the cancer cells in PSM or medium overnights until the final cell viability assay. PSM: CAP-stimulated medium. PSB: CAP-stimulated simple buffered solution.

Compared with the abundant studies on the direct CAP treatment, the understanding of the anti-cancer effect of PSS is lacking. To date, only four biologically adaptable solutions have been used as PSS. Most studies used cell culture medium to make PSS^13, 16–30, 34–36^. Few studies used simple buffered solutions such as phosphate buffered solution (PBS)^24, 36, 37^ and Lactated Ringer’s solution^30^ to make PSS. To investigate the anti-cancer capacity of PSS *in vitro*, at least three research strategies have been proposed (Fig. 1). In this study, the CAP-stimulated medium and the CAP-stimulated simple buffered solution is designated as PSM and PSB, respectively. Considering the fact that medium as the control is suitable for cell culture over a long time, PSM can be used to culture cancer cells overnight^13, 16–30, 34–36^. Whereas, the simple buffered solutions without the necessary nutrients can be just used to affect the cancer cells for a relative short time such as several hours. At least two research strategies have been developed for PSB. One is directly using PSB to affect cancer cells for a short time length such as several hours^30^. After that, PSB is replaced by the untreated medium. The cancer cells will be cultured in the new medium overnight until the final cell viability assay or other analysis is performed. Another strategy is using the mixture of PSB and the untreated medium to culture cells overnight. In this case, despite the medium will be diluted by PSB, its nutrient will still sustain the growth of cancer cells overnight^24^.

The understanding of the anti-cancer mechanism of PSM has been extensively investigated. The CAP-original reactive species such as H_2_O_2_^18, 20–22, 24, 26, 34^, NO_2_^−^^27, 37^, as well as NO_3_^−^^27, 37^ have been regarded as the key anti-cancer species in PSM. Catalase or other simple chemicals such as cysteine and methionine can effectively eliminate the anti-cancer capacity of PSM through consuming H_2_O_2_^20, 21, 38^. Thus, H_2_O_2_ may be a key anti-cancer species formed in PSM. H_2_O_2_ can cause DNA damage on cancer cells, which is mediated by hydroxyl radicals generated from H_2_O_2_ by the Fenton reaction^39^. H_2_O_2_ is also an efficient inductor for the apoptosis in cancer cells^39, 40^. H_2_O_2_-generating drugs can effectively kill cancer cells by the increase of cellular levels of H_2_O_2_^39^. Experimental evidences demonstrate that cancer cells are more susceptible to H_2_O_2_ with specific concentration than normal cells^39, 41, 42^. Moreover, increasing the concentration of reactive species in PSM by chemical methods^20, 24^ or physical methods^21^ is an effective strategy to optimize the anti-cancer capacity of PSM.

Whereas, the understanding on the anti-cancer mechanism of PSB is just limited to few refs 24, 30, 36 and 37. The anti-cancer capacity of PSB is highly affected by the research strategies chosen by authors. Compared with PSM, the killing effect of PSB is always weaker. As early as in 2011, Sameer Kalghatgi, *et al*. first found that noticeable double strand DNA breaks just occurred in the CAP treatment on medium rather than on PBS^43^. Very recently, Hiromasa Tanaka, *et al*. found that the CAP-treated water could not cause the noticeable killing effect on glioblastoma cells (U251SP)^30^. Corresponding noticeable rise of intracellular reactive oxygen species (ROS) just occurred in the cells cultured in PSM rather than in PSB^30^. In fact, the H_2_O_2_ generation in PSM and PSB are similar in these cases^30^. These contradictions indicate that just the reactive species in PSB may not necessarily result in a noticeable damage on cancer cells. However, it is widely observed that the reactive species in PSM would definitely cause noticeable cancer cells death^13, 16–30, 34–36^. In addition, we have found that using the PSB/untreated DMEM mixture could also achieve a significant anti-cancer effect over glioblastoma cells (U87MG), pancreatic adenocarcinoma cells (PA-TU-8988T), as well as breast adenocarcinoma cells (MDA-MB-231). In short, the anti-cancer species may be toxic to cancer cells just in PSM or PSB/medium mixture rather than in PSB. The unique characteristics differentiating PSM and PSB, and their subsequent interaction on cancer cells have yet to be elucidated.

In this study, we systematically compared the anti-cancer effect of PSM and PSB on two cancer cell lines, U87MG and PA-TU-8988T. We identified that the effect on cancer cells is cell-dependent and dose-dependent. Both PSM and PSB could inhibit the growth of cancer cell lines when the right experimental conditions were set. U87MG cells and PA-TU-8998T cells were highly vulnerable to PSB and PSM, respectively. The measurement of intracellular ROS of two cell lines in PSM and PSB first revealed that the different cellular responses in medium and in PBS might cause the different vulnerabilities of cells to PSM and PSB.

## Methods

### CAP device

The CAP jet device was designed and assembled in our lab and has been used in a series of studies about the anti-cancer capacity of the CAP treatment *in vitro* ^19, 21, 25, 44, 45^ and *in vivo* ^10^. It used helium as the carrying gas. The CAP jet was formed by the discharge between a ring grounded cathode and a central anode and was flowed out (4.7 L/min) the quartz tube with a diameter of 4.5 mm. The input voltage of DC power was 11.5 V. The output voltage was 3.16 kV. The plasma discharge was driven by an AC high voltage with a frequency of 30 kHz.

### Cells culture

Human glioblastoma (U87MG) cells and human pancreas adenocarcinoma (PA-TU-8988T) cells were provided by Dr. Murad’s lab at the George Washington University. Human breast adenocarcinoma cells (MDA-MB-231) were provided by Dr. Zhang’s lab at the George Washington University, and were cultured in the same protocol as previous studies^46, 47^. The Dulbecco’s modified Eagle’s medium (DMEM, with L-glutamine, 11965–118) was purchased from Life Technologies. DMEM was mixed with 1% (v/v) antibiotic (penicillin and streptomycin) solution (Life Technologies). The media used in cell culture were composed of DMEM supplemented with 10% (v/v) fetal bovine serum (ThermoFisher Scientific). All wells on the margins of a 96-wells plate (61406–081, Corning) were not used in this study. Only 6 wells in a single column on a 96-wells plate were used to seed cells. 100 μL of the cells-harvesting solution (6 × 10^4^ cells/mL) were seeded in each well. Cancer cells were grown for 1 day under the standard culture condition (a humidified, 37 °C, 5% CO_2_ environment). The media that have been used to culture cells overnight were removed before using PSM or PSB to treat cancer cells.

### Extracellular H_2_O_2_ assay

The H_2_O_2_ concentration in sample solutions was measured by using Fluorimetric Hydrogen Peroxide Assay Kit (Sigma-Aldrich) following the protocols provided by manufacturer. Finally, 50 μL of H_2_O_2_ probe solution was mixed with 50 μL of sample in the black 96-wells plate. After 30 min of storage at the room temperature without the ambient light, the fluorescence was measured by a H1 microplate reader (Hybrid Technology) at 540/590 nm. The final fluorescent strength of the experimental group was obtained by deducting the fluorescence of the control group from the fluorescence of the experimental group. The H_2_O_2_ concentration in the samples were calculated based on the standard curve. In each experiment, the sample number was 3. The average of 3 samples was set as one result.

### Making (N-acetyl-L-cysteine) NAC containing DMEM (NAC-DMEM) and pre-treating cancer cells

6 mM and 10 mM NAC-DMEM were made by dissolving NAC powder (A7250, Sigma-Aldrich) in DMEM. As an intracellular ROS scavenger, NAC will penetrate the cytoplasmic membrane and resist the redox balance change due to the rise of intracellular ROS^13, 17, 48^. To pre-treat cancer cells with NAC-DMEM, the medium which has been used to culture cancer cells overnight were removed first. Then, 100 μL of NAC-DMEM were used to culture the cancer cells in each well of a 96-wells plate. After 3 hr, the NAC-DMEM were removed. These cancer cells were ready for the further treatment.

### Making PSM/PSB, H_2_O_2_-containing DMEM/PBS (H_2_O_2_-DMEM/PBS), NO_2_^−^-containing DMEM (NO_2_^−^-DMEM) and treating cancer cells

In this study, Lonza BioWhittaker Dulbecco’s PBS did not contain calcium and magnesium (BW17512F12, Fisher Scientific). To make PSM/PSB, 1 mL of DMEM in a well of a 12-wells plate (61406–165, Corning) was treated by CAP. The gap between the bottom of 12-wells plate and the CAP source was 3 cm. To make H_2_O_2_-DMEM/PBS, 9.8 M H_2_O_2_ standard solution (216763, Sigma-Aldrich) were added in DMEM or PBS with a designed concentration. Similarly, to make NO_2_^−^-DMEM, 0.1 M nitrite ion standard solution (72586, Sigma-Aldrich) was added in DMEM with a designed concentration. To affect the growth of cancer cells, 100 μL of the prepared solutions including PSM/PSB, H_2_O_2_-DMEM/PBS, NO_2_^−^-DMEM was transferred to affect cancer cells grown on a 96-wells plate immediately after the preparation. For the control group, cancer cells were just cultured in the untreated DMEM or in the untreated PBS. In each experiment, the sample number was 6. For the experiment using the PBS-based solutions, cancer cells were just cultured in the CAP-stimulated PBS for 3 hr. The new DMEM replaced the CAP-stimulated PBS after that and further sustained the culture for 1 day. For the experiment using DMEM, cancer cells were cultured for 1 day after the treatment. These sextuplicate experiments were independently repeated for at least twice.

### Measuring the H_2_O_2_ consumption speed by cancer cells

The protocols for different cancer cell lines were identical. Here, we used U87MG cells as an example. First, 100 μL of cells harvesting solution with a confluence of 6 × 10^4^ cells/mL were seeded in a well on a 96-wells plate. In each experiment, 3 wells on 96-wells plate were seeded with cells as 3 samples. Cells were cultured in the incubator for 7 hr under the standard conditions. Then, 1 mL of DMEM or PBS in a well on a 12-wells plate was treated by CAP for 1 min. After that, 100 μL of PSM or PSB was transferred to culture cells grown on a 96-wells plate. The medium which has been used to culture cells was removed before this step. Since then, 50 μL of medium which has been used to culture cells was transferred to a well on a black clear bottom 96-wells plate (29444–008, Corning) in triplicate every hour until the third hour. Ultimately, the residual H_2_O_2_ concentration in PSM or PSB was measured using fluorimetric assay illustrated above.

### Making the diluted PSM and affecting cancer cells

We prepared a series of diluted DMEM by gradually increasing the dilution fraction of PBS in DMEM from 0% to be 90%. The corresponding dilutability of DMEM gradually decreased from 0% to be 90%. Because cancer cells needed to be cultured overnight, we have not performed the experiment with a 100% of dilutability. Then, 1 mL of DMEM with different dilutabilities in a well of 12-wells plate (61406–165, Corning) was treated by CAP. The gap between the bottom of 12-wells plate and the CAP source was 3 cm. To affect the growth of cancer cells, 150 μL of PSM with different dilutabilities was transferred to affect cancer cells grown on a 96-wells plate immediately. In control groups, cancer cells were just cultured in the untreated DMEM with different dilutabilities. In each experiment, the sample number was 6. Cancer cells were cultured for 1 day before the cell viability assay. All experiments were independently repeated for at least 3 times in sextuplicate.

### Cell viability assay

The cell viability was measured by using MTT (3-(4,5-Dimethyl-2-thiazol)-2,5-Diphenyl-2H-tetrazolium Bromide) assay following the standard protocols provided by manufacturer (M2128, Sigma-Aldrich). The 96-wells plate was read by a H1 microplate reader (Hybrid Technology) at an absorbance of 570 nm. To facilitate the formation of final violet solution, the 96-wells plates were shook for 30 s before reading. To facilitate the data analysis, the measured absorbance at 570 nm was processed to be a relative cell viability by the division of absorbance between the experimental group and the control group. In each experiment, the cell viability was equal to the mean of 6 samples from 6 wells.

### Measuring intracellular ROS

The cell permeant 2,7-dichlorodihydrofluorescein diacetate (CM-H2DCFDA), a general oxidative stress indicator (ThermoFisher Scientific) has been widely used as the intracellular ROS probes in plasma medicine, though the determination of intracellular ROS by these probes may be influenced by the level of free cytochrome c in cells^49^. 500 mL of 1× buffer (113851, Abcam) were added into the tube containing 50 μg of molecular probes CM-H2DCFD to prepare the 1× stock solution. 1× stock solution was further diluted to be 0.03125× stock solution. Prior to the measurement, 100 μL of 25 × 10^4^ cells/mL cells were seeded in the well on a black clear bottom 96-wells plate (29444–008, Corning) and were cultured for 1 day. For each case, the samples in experimental group and control group were all 3. The controls groups and the experimental groups were the measurements performed on cancer cells just cultured in the untreated DMEM/PBS and in the H_2_O_2_-DMEM/PBS, respectively. The 10 min- and 20 min-equivalent H_2_O_2_-DMEM/PBS were prepared by adding 30 wt % (9.8 M) H_2_O_2_ solution (216763, Sigma-Aldrich) in DMEM/PBS. The concentration of 10 min- and 20 min-equivalent H_2_O_2_-DMEM/PBS were 367.5 μM and 735 μM, respectively. The intracellular ROS measurement of PA-TU-8988T cells and U87MG cells began 3 hr and 50 min after the H_2_O_2_ treatment on cells, respectively. Cells were washed by 100 μL of 1× buffer. Then, cells were cultured in 100 μL of 0.03125× stock solution for 50 min. Subsequently, 100 μL of PBS was used to replace the stock solution in each well. Finally, the fluorescence was measured by a H1 microplate reader (Hybrid Technology) at 485/535 nm. For the data processing, the measured fluorescence of the control groups and the experimental groups were first subtracted by the fluorescence of background (just PBS). The fluorescence of the experimental group was modified by subtracting the fluorescence of the control group. Ultimately, the modified fluorescence of the experimental groups was modified to be a relative intracellular ROS based on the following formula. Relative intracellular ROS = Modified experimental groups/Modified control group.

### Data availability statement

All data generated or analysed during this study are included in this published article (and its Supplementary Information files).

### Experiments and results

**Figure 2:**
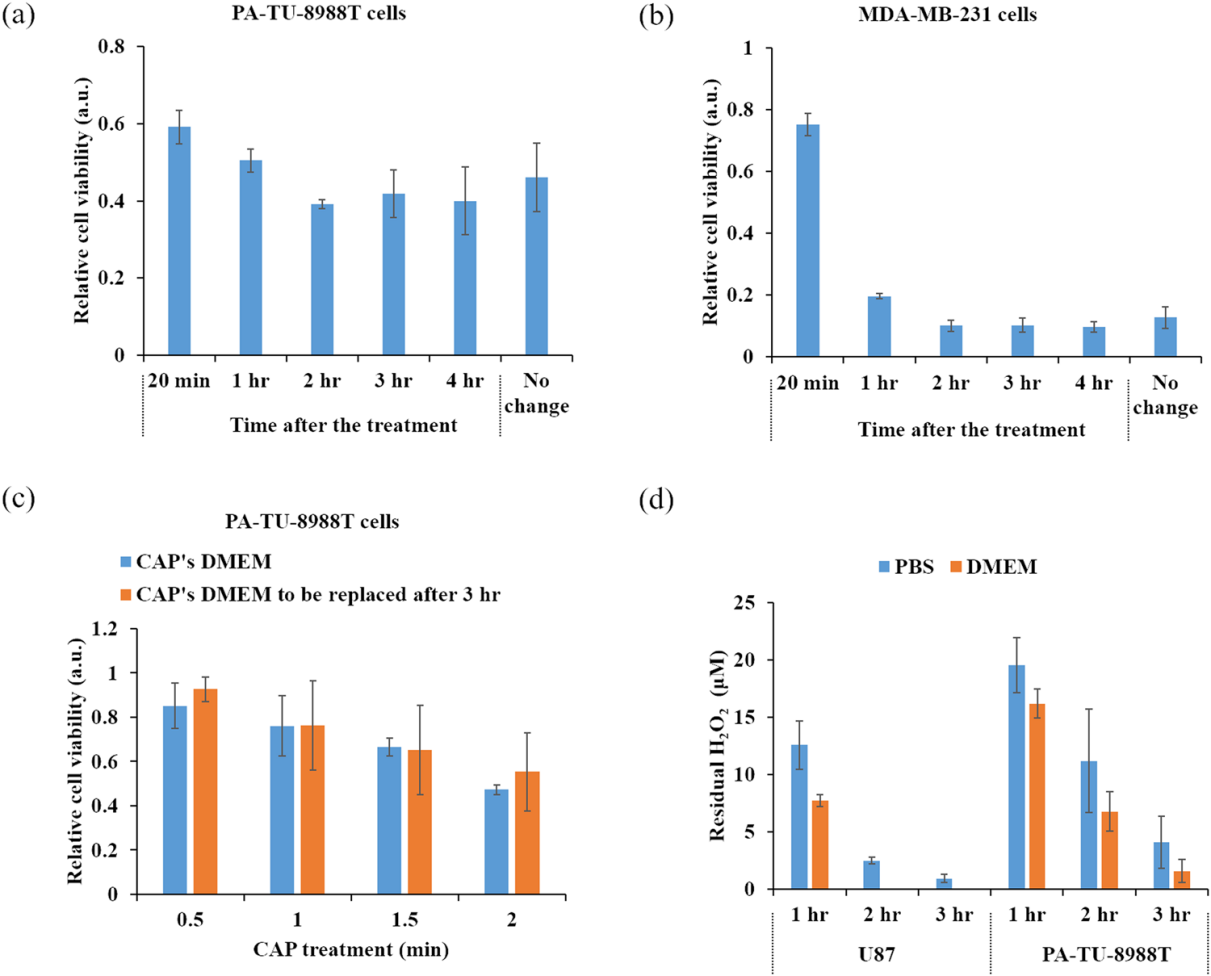
The initial several hours after the treatment are key for the anti-cancer effect of PSS. After the treatment for 2 to 4 hr, removing the PSM from cancer cells’ environment would not affect the anti-cancer effect on PA-TU-8988T cells (**a**) and MDA-MB-231 cells (**b**). No change means that PSM were used to culture cancer cells for 1 day without renewing the medium. (**c**) After the treatment for 3 hr, replacing the CAP-stimulated (CAP’s) DMEM by the untreated DMEM just caused a similar anti-cancer effect on PA-TU-8988T cells as using the CAP’s DMEM to affect cells did. (**d**) The extracellular H_2_O_2_ were completely consumed by cancer cells in just 3 hr after the treatment. Results in (**a**), (**b**) and (**c**) are presented as the mean ± s.d. of two independently repeated experiments performed in sextuplicate. Results in (**d**) are presented as the mean ± s.d. of two independently repeated experiments performed in triplicate.

PBS cannot be used to culture cancer cells for a relative long time. In the first study about the toxicity of PSB on mammalian breast epithelial cells (MCF10A), the cells were just immerged in PBS for a short time length^43^. In this study, PSB was only used to affect the growth of cancer cells for several hours before we used the untreated DMEM to replace PSB and further sustain the culture overnight. Finding a proper culture time for the cancer cells in PSB is necessary for an effective comparison with PSM. In plasma, the common way to use PSM is just culturing cells in PSM overnight or for several days (Fig. 1). Thus, we first investigated whether the initial several hours of culture were important for the toxicity of PSM on PA-TU-8988T cells. Specifically, PA-TU-8988T cells grown on a 96-wells plate were cultured for 24 hours. Following the protocols in methods about making PSM, 1 mL of PSM were made by a 3 min of CAP treatment on DMEM. 100 μL of PSM was transferred to affect the growth of PA-TU-8988T cells immediately. Afterward, 100 μL of new DMEM replaced PSM to culture cancer cells and changed every hour after the treatment until the fourth hour (Fig. 2a). Finally, PA-TU-8988T cells were cultured for 24 hours followed by the cell viability assay according the protocols in Methods. For MDA-MB-231 cells, the protocols were similar except the CAP treatment was performed on 6-wells plate for 1 min with the same changing of media as above.

It is found that removing the PSM which has been cultured with the cancer cells for 2 to 4 hr could not change the toxicity of PSM on MDA-MB-231 cells and PA-TU-8988T cell viability (Fig. 2a and b). The noticeably weakened anti-cancer capacity only occurred when PSM was removed prior to 1 hr after the initiation of treatment (Fig. 2a and b). To further confirm this trend, we investigated the anti-cancer capacity of PSM on PA-TU-8988T cells by replacing PSM with new DMEM 3 hr after using PSM to affect cells. PSM was made by treating 1 mL of DMEM in a 12-wells plate with CAP. Compared with the case that just culturing PA-TU-8988T cells in PSM for 1 day, removing PSM from the culturing environment of PA-TU-8988T cells just 3 hr after the treatment, did not produce noticeable difference in terms of anti-cancer capacity (Fig. 2c).

These observations indicate that the anti-cancer reactive species in PSM has been significantly consumed in the initial several hours after the CAP treatment. Thus, we further investigated the residual H_2_O_2_ concentration in PSM and PSB which was used to culture cancer cells for several hours. The protocols are illustrated in Methods. We identified a significant consumption of H_2_O_2_ in PSM by the two cancer cell lines in the initial 3 hr (Fig. 2d). U87MG cells consumed H_2_O_2_ faster than PA-TU-8988T cells did. In addition, the two cancer cell lines consumed H_2_O_2_ at slightly faster rate in DMEM than that in PBS (Fig. 2d). The H_2_O_2_ in PSM has been completely consumed by U87MG cells just 2 hr after the treatment. The residual H_2_O_2_ in PSM and PSB have nearly been completely consumed by two cell lines just in the initial 3 hr after the treatment. This result is consistent with the observation that replacing PSM by new DMEM 3 hr after the treatment will not affect the anti-cancer capacity of PSM.

**Figure 3:**
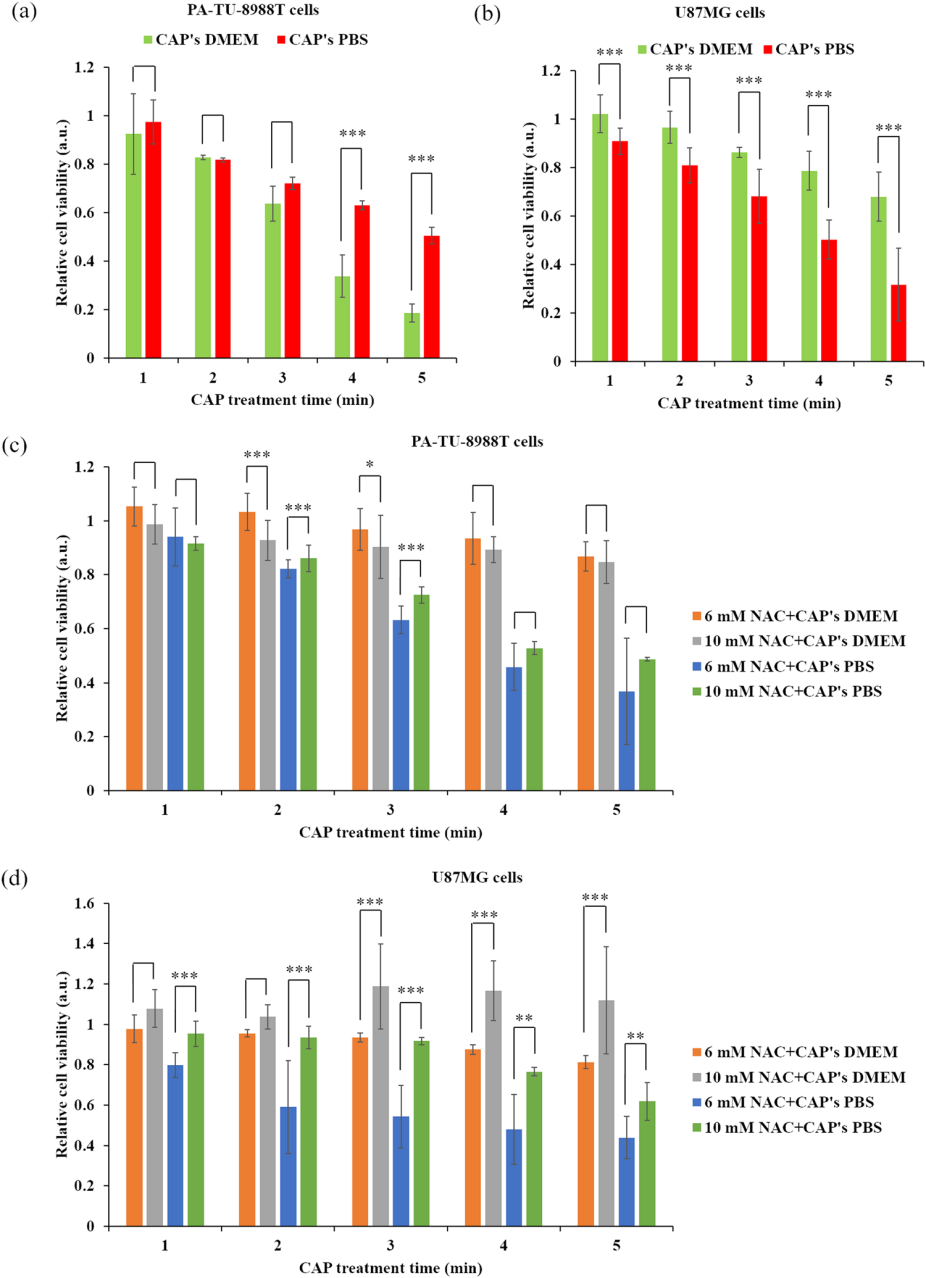
The different toxicities of PSM/PSB on cancer cells. (**a**) PA-TU-8988T cells. (**b**) U87MG cells. (**c**) Pre-treating PA-TU-8988T cells with NAC. (**d**) Pre-treating U87MG cells. As we illustrated in Methods, PSB was just used to culture cells for 3 hr before using new DMEM to replace it. Results are presented as the mean ± s.d. of two independently repeated experiments performed in sextuplicate. Student’s t-test was performed and the significance is indicated as ***p < 0.005, **p < 0.01, and *p < 0.05.

To compare the anti-cancer capacity of PSM and PSB, DMEM and PBS were treated by the CAP jet under the same experimental conditions. The killing effect of PSM and PSB on cancer cells will be noticeable as long as the treatment time was longer than 2 min. PSM was much more toxic to PA-TU-8988T cells than PSB when the CAP treatment time was longer than 3 min (Fig. 3a). In contrast, PSB was much more toxic to U87MG cells than PSM when the CAP treatment time was longer than 2 min (Fig. 3b). Pre-treating cancer cells with the intracellular ROS scavengers such as D-mannitol^48^, rotenone^50^, apocynin^50^, as well as NAC^13, 48, 51^ can drastically counteract the anti-cancer effect of CAP treatment. We further investigated the effect of NAC on the anti-cancer effect of PSM and PSB. As shown in Fig. 3c and d, both 6 mM and 10 mM NAC could completely inhibit the killing effect of PSM in the two cell lines. Particularly, for U87MG cells, 10 mM NAC resulted in a noticeably higher cell viability than 6 mM NAC does (Fig. 3d). On the contrary, the pre-treatment of NAC showed remarkably different effects on the toxicity of PSB between two cell lines. For the PSB-treated U87MG cells, 6 mM NAC did not cause a noticeable increase on the cell viability (Fig. 3d). However, 10 mM NAC significantly increased the cell viability about 100% compared the cells treated by the PSB made by 5 min of CAP treatment. (Fig. 3d). For PA-TU-8988T cells, both 6 mM and 10 mM NAC could not noticeably increase the viability of the PSB-treated cancer cells (Fig. 3c). Based on these results, it appears that the two cell lines experience different cellular responses in PSM and in PSB that lead to the decrease of tumor cell viability with different levels.

**Figure 4:**
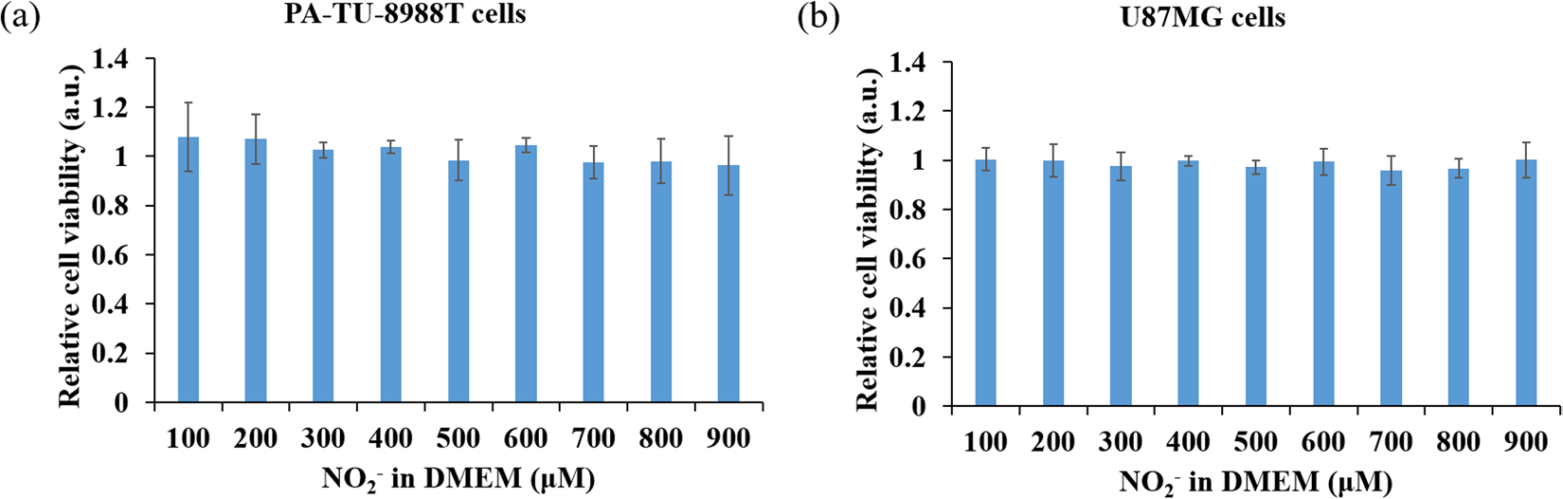
The toxicity of NO_2_^−^ on cancer cells. (**a**) PA-TU-8988T cells. (**b**) U87MG cells. Results are presented as the mean ± s.d. of three independently repeated experiments performed in sextuplicate.

The CAP-originated reactive species including ROS and RNS have been regarded as the main anti-cancer factors during the CAP treatment *in vitro* ^5^. Despite many researchers regard H_2_O_2_ as the main anti-cancer species generated by the CAP treatment^18, 20–22, 24, 26, 34, 52–54^, it is still disputable that RNS might be a key anti-cancer species^55^. We analyzed the role of NO_2_^−^, a widely confirmed CAP-originated RNS product^53, 56, 57^ in the anti-cancer capacity of PSM. The NO_2_^−^concentration in PSM made by 1 min of CAP treatment was approximately 10 μM. The toxicity on cancer cells of concentrations ranging from 100 μM to 900 μM NO_2_^−^-DMEM on PA-TU-8988T cells, U87MG cells, as well as MDA-MB-231 cells were studied. Clearly, even 900 μM NO_2_^−^-DMEM which was equivalent to a PSM experienced about 90 min of CAP treatment would not noticeably inhibit the growth of all three cell lines at all (Figs 4 and S1). Thus, NO_2_^−^ at least does not independently contribute to the toxicity of PSM to cancer cells.

**Figure 5:**
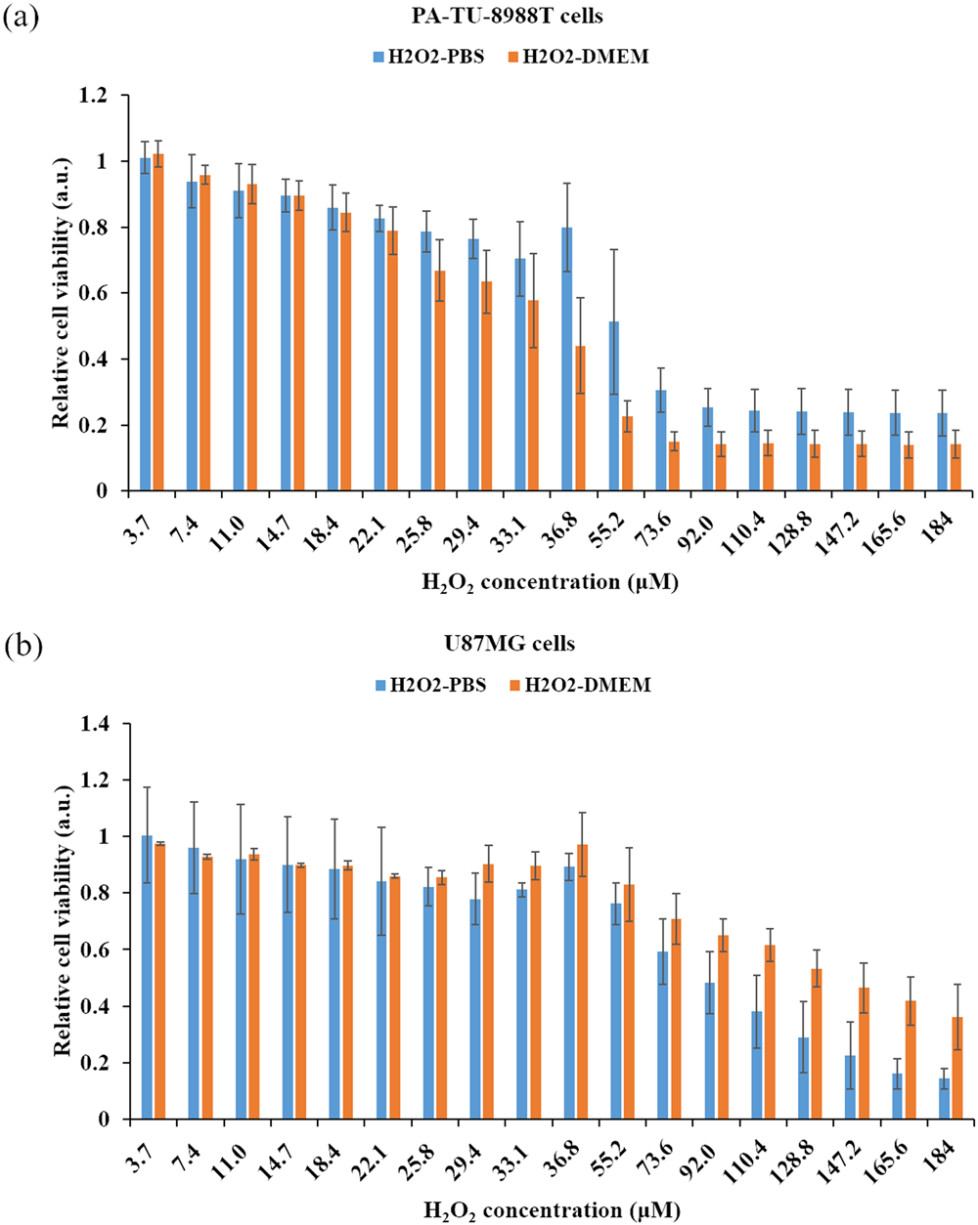
The toxicity of the H_2_O_2_-DMEM and the H_2_O_2_-PBS on cancer cells. (**a**) PA-TU-8988T cells. (**b**) U87MG cells. As we illustrated in Methods, H_2_O_2_-PBS was just used to culture cells for 3 hr before using new DMEM to replace it. 36.8 μM of H2O2-PBS/DMEM is the equivalent solution of 1 min of CAP-stimulated PBS/DMEM. Results are presented as the mean ± s.d. of three independently repeated experiments performed in sextuplicate.

The toxicity of H_2_O_2_-DMEM and H_2_O_2_-PBS on PA-TU-8988T cells and U87MG cells were also investigated. We identified that 1 min of CAP treatment in DMEM or PBS would generate 36.3 μM H_2_O_2_. Thus, 36.3 μM H_2_O_2_-DMEM/PBS was the 1 min- equivalent CAP-stimulated DMEM/PBS. As shown in Fig. 5, the vulnerability of the two cell lines to H_2_O_2_ was also cell-dependent. For PA-TU-8988T cells, when the H_2_O_2_ concentration was larger than 36.3 μM, H_2_O_2_-DMEM has significantly higher toxicity to cancer cells than H_2_O_2_-PBS. On the contrary, when the H_2_O_2_ concentration was larger than 72.6 μM, H_2_O_2_-PBS there was significantly more toxicity to for U87MG cells than H_2_O_2_-DMEM. Such cell-dependent difference is well consistent with the trends obtained from experiments based on PSS (Fig. 3), which again confirms that H_2_O_2_ is the main anti-cancer species in PSS compared with NO_2_^−^.

**Figure 6:**
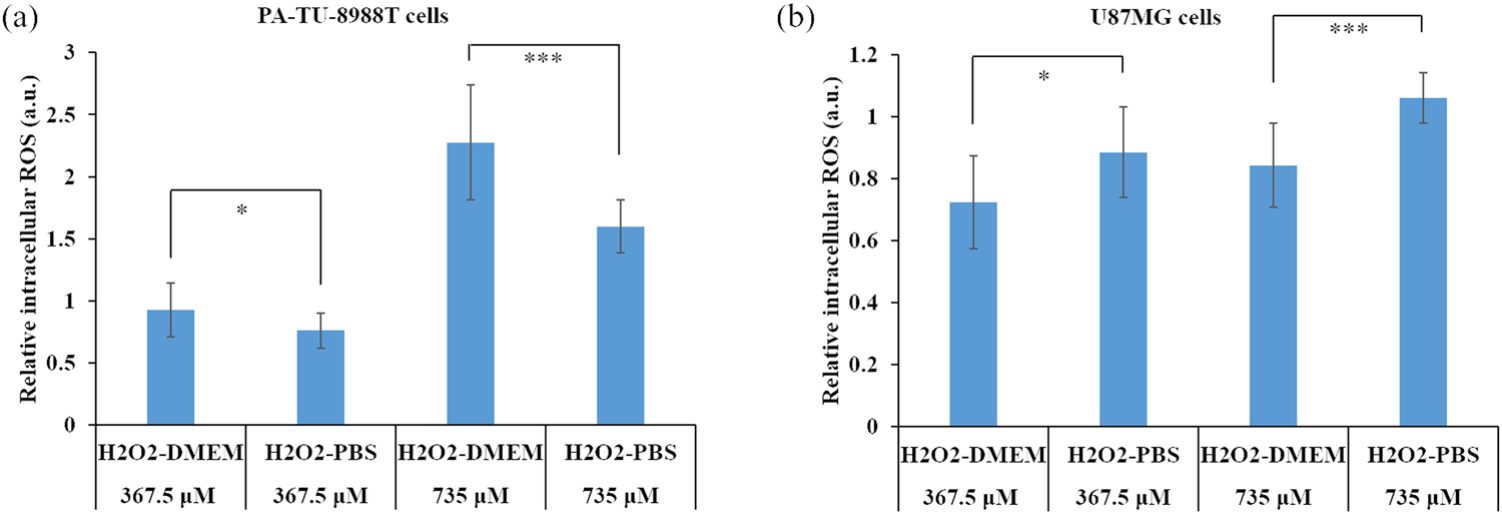
The different intracellular ROS changes due to the H_2_O_2_ treatment. (**a**) PA-TU-8988T cells. (**b**) U87MG cells. Results are presented as the mean ± s.d. of two independently repeated experiments in triplicate. Student’s t-test was performed and the significance is indicated as ***p < 0.005, **p < 0.01, and *p < 0.05.

The rise of intracellular ROS is regarded as a key cellular response to the CAP treatment^5, 58^. The disturbed redox balance^31, 54^, serious DNA damage^13, 59, 60^, as well as mitochondria damage^15, 48, 61^ may be all caused by the rise of intracellular ROS. H_2_DCFDA is a common intracellular ROS probe used in plasma medicine^13, 17, 48, 51, 52, 56^. In this study, we also used H_2_DCFDA as the probe. Following the protocols provided by the manufacturer, the intracellular ROS measurement needs a high cell confluence (25 × 10^4^ cells/mL). Because the cellular response to the CAP treatment is highly confluence-dependent^21, 24^, such high confluence needs a very high reactive species concentration to generate an observable cellular response. Thus, 10 min-equivalent (367.5 μM) and 20 min-equivalent (735 μM) H_2_O_2_-rich DMEM/PBS were used to simulate the response of cancer cells to the PSS with a similar H_2_O_2_ concentration. For PA-TU-8988T cells, H_2_O_2_-DMEM caused a stronger intracellular ROS rise than that seen in H_2_O_2_-PBS (Fig. 6a). For U87MG cells, H_2_O_2_-PBS caused a stronger intracellular ROS rise than that seen in H_2_O_2_-DMEM (Fig. 6b). The trends observed in the rise of intracellular ROS explain the trends observed in the anti-cancer capacity of PSS (Figs 3a and b). The noticeable rise of intracellular ROS in the PSM-treated cancer cells rather than in the PSB-treated cancer cells has also been observed recently^30^. Based on all above results, it is reasonable to conclude that the different toxicities of PSS on different cancer cell lines is at least partially due to the different rise of intracellular ROS in different cancer cells exposed to the reactive species particularly H_2_O_2_ in PSS. An optimized strategy for using PSM and PSB in cancer treatment should therefore be cell-dependent.

**Figure 7:**
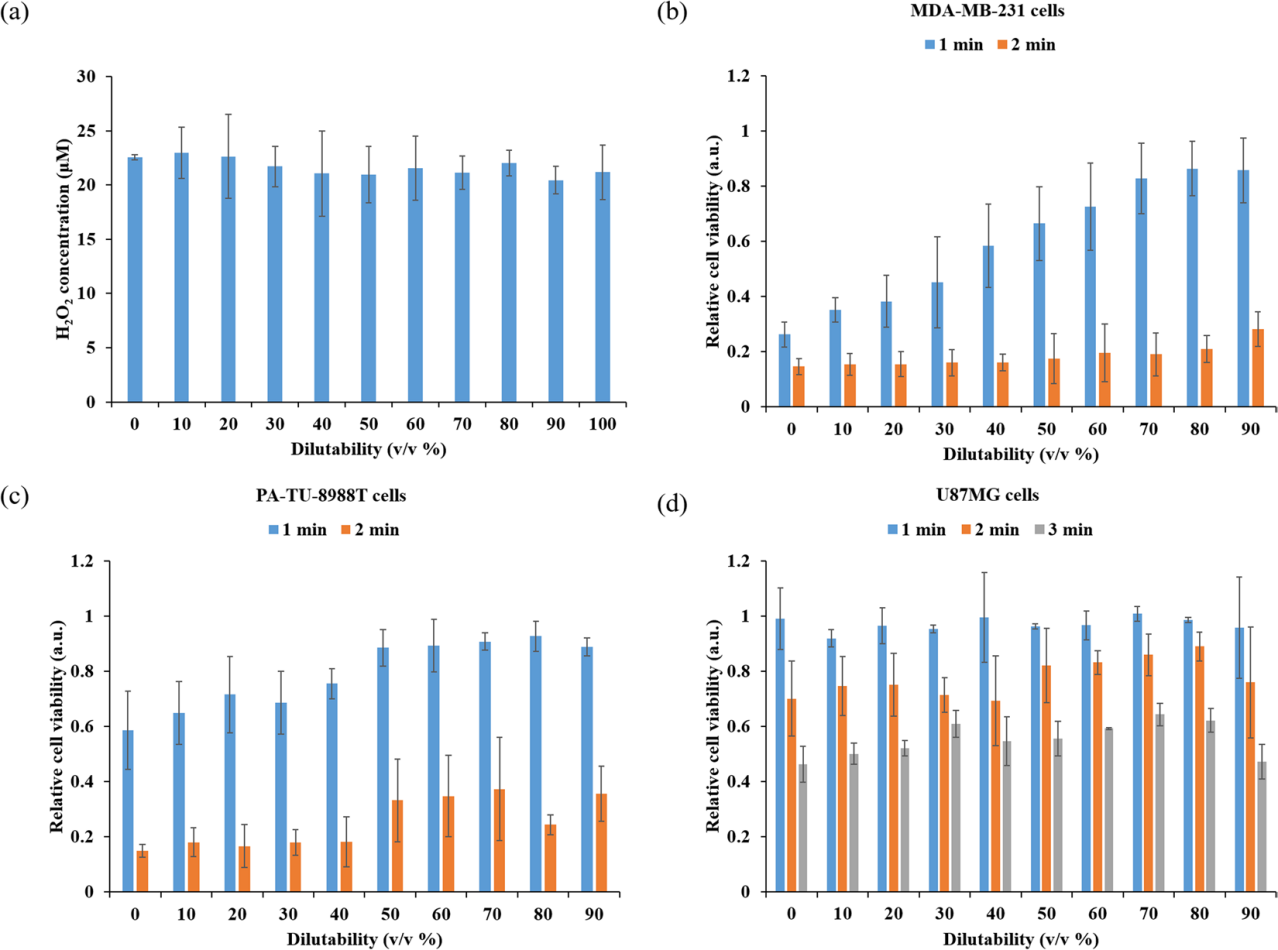
The dilution in medium by PBS significantly weakens the anti-cancer capacity of PSS in some cancer cell lines. (**a**) The concentration of H_2_O_2_ in the PSM with different dilutabilities. (**b**) Breast adenocarcinoma cell line (MDA-MB-231). (**c**) Pancreas adenocarcinoma cell line (PA-TU-8988T). (**d**) Glioblastoma cell line (U87MG). 0% of dilutability and 90% of dilutability represents a mixed DMEM composed of 0% (v/v) PBS + 100% (v/v) DMEM and a mixed DMEM composed of 90% (v/v) PBS + 10% (v/v) DMEM, respectively. Result in (**a**) presented as the mean ± s.d. of three independently repeated experiments performed in triplicate. Results in (**b**,**c** and **d**) presented as the mean ± s.d. of three independently repeated experiments performed in sextuplicate.

In fact, DMEM a solution with abundant nutrient and PBS a solution with few nutrients should be regarded as the extreme cases in biologically adaptable solutions. We further identified a general trend for other biological adaptable solutions through investigating the dilution effect of PBS in DMEM on the anti-cancer effect of PSS. Following the protocols in Methods, a series of diluted DMEM solutions were made by increasing the volume fraction of PBS in DMEM from 0% (v/v) to be 90% (v/v). The volume fraction is also named as the dilution fraction or the dilutability in this study. The dilution effect on the killing effect of PSM over MDA-MB-231 cells, PA-TU-8988T cells, as well as U87MG cells were studied. Despite the dilution on DMEM did not change the H_2_O_2_ concentration in PSS (Fig. 7a), the dilution effect showed a remarkable impact on the anti-cancer capacity of PSS. For MDA-MB-231 cells and PA-TU-8988T cells, the effect of cancel cell viability drastically decreased as the dilutability in DMEM increased when the treatment time was 1 min (Fig. 7b and c). In other words, the dilution in DMEM protected these cell lines from the attack of reactive species. When the treatment time increases to 2 min, a noticeable increased anti-cancer effect is obtained in all experiments with different dilution levels. The trend that the CAP-stimulated highly diluted DMEM is more resistant to the effect of reactive species still exists (Fig. 7b and c). These results indicate that the dilution effect may change the threshold of cancer cells to the effect of the CAP-originated species, rather than a difference in the actual CAP-originated species generated (Fig. 7a). For U87MG cells, the dilution effect exerts a similar but a weaker dilution effect even up to a CAP treatment time of 3 min (Fig. 7d). Such a difference may be due to the much stronger resistance seen in U87MG cells to the CAP treatment compared with PA-TU-8988T cells and MDA-MB-231 cells^21, 24^. However, when the dilutability increases from 80% to 90%, the killing effect of PPS on U87MG cells is decreased. In short, the dilution effect as a new factor can drastically regulate the anti-cancer capacity of PSS.

## Discussion

The anti-cancer capacity of PSS mainly PSM has been demonstrated over the past 5 years. From these studies, the reactive species H_2_O_2_, NO_2_^−^, as well as NO_3_^−^ have been regarded as the main factors affecting the death of cancer cells^18, 20–22, 24, 26, 27, 34, 37^. On the contrary, the biologically adaptable buffered solution such DMEM and PBS have just been regarded as a carrier for the dissolved CAP-originated reactive species^16–28, 34–37^. Such understanding can satisfactorily explain the common observation that PSM, the most commonly investigated PSS, is an effective anti-cancer tools on several cell lines *in vitro*. This explanation conflicts with the observation that directly using PSB to affect cells will just generate a much weaker and perhaps a negligible killing effect compared with using PSM^30, 43^.

In this study, through a comprehensive investigation on the anti-cancer effect of PSM and PSB on pancreatic adenocarcinoma cells and glioblastoma cells, and the rise of intracellular ROS in these cells upon the treatment of PSM and a PSB, we can give a comprehensive explanation to previous conflicting observations. First, the concentration of the main toxic reactive species H_2_O_2_ in PSM and PSB is the same (Fig. 6a). Second, both PSM and PSB are toxic to cancer cells as long as the CAP treatment time is adequately long, such as 4–5 min revealed in this study (Fig. 3a and b). Particularly for the experiment using H_2_O_2_-DMEM or H_2_O_2_-PBS, their strong toxicities on pancreatic adenocarcinoma cells and glioblastoma cells clearly demonstrate the reactive species such as H_2_O_2_ can kill cancer cells culture in DMEM or PBS (Fig. 5a and b). Thus, the earliest observation that the mammalian cells immersed in PBS did not experience DNA damage upon the CAP treatment may be partially due to a relatively weak CAP treatment dose^43^. Based on this study, it is reasonable to speculate that serious DNA damage and apoptosis in cancer cells will also occur as long as the CAP treatment is adequately long even if just PSB has been used to affect cancer cells.

More importantly, the anti-cancer effect of PSM/PSB is cell-dependent (Fig. 3a and b). Specifically, PSM is much more toxic than PSB on PA-TU-8988T cells. On the contrary, PSB is much more toxic than PSM on U87MG cells. And, this trend will not be changed if these cells are just treated by H_2_O_2_-DMEM and H_2_O_2_-PBS (Fig. 5a and b). Thus, such specific cellular response of cancer cells to PSM or PSB is at least partially determined by the specific cellular response of cancer cells in H_2_O_2_-containing environment. In addition, we identified that the rise of intracellular ROS upon the treatment of H_2_O_2_-DMEM and H_2_O_2_-PBS is also cell-dependent (Fig. 6a and b). Thus, the stronger rise of intracellular ROS in one cancer cell line than another cell line is also determined by the specific response of cancer cells to reactive species such as H_2_O_2_ in a specific extracellular environment such as DMEM or PBS. Based on our findings, it is reasonable to speculate that the previous conflicting observations about the stronger anti-cancer effect of PSM compared with PSB may just reflect one component of the whole picture revealed in this study.

**Figure 8:**
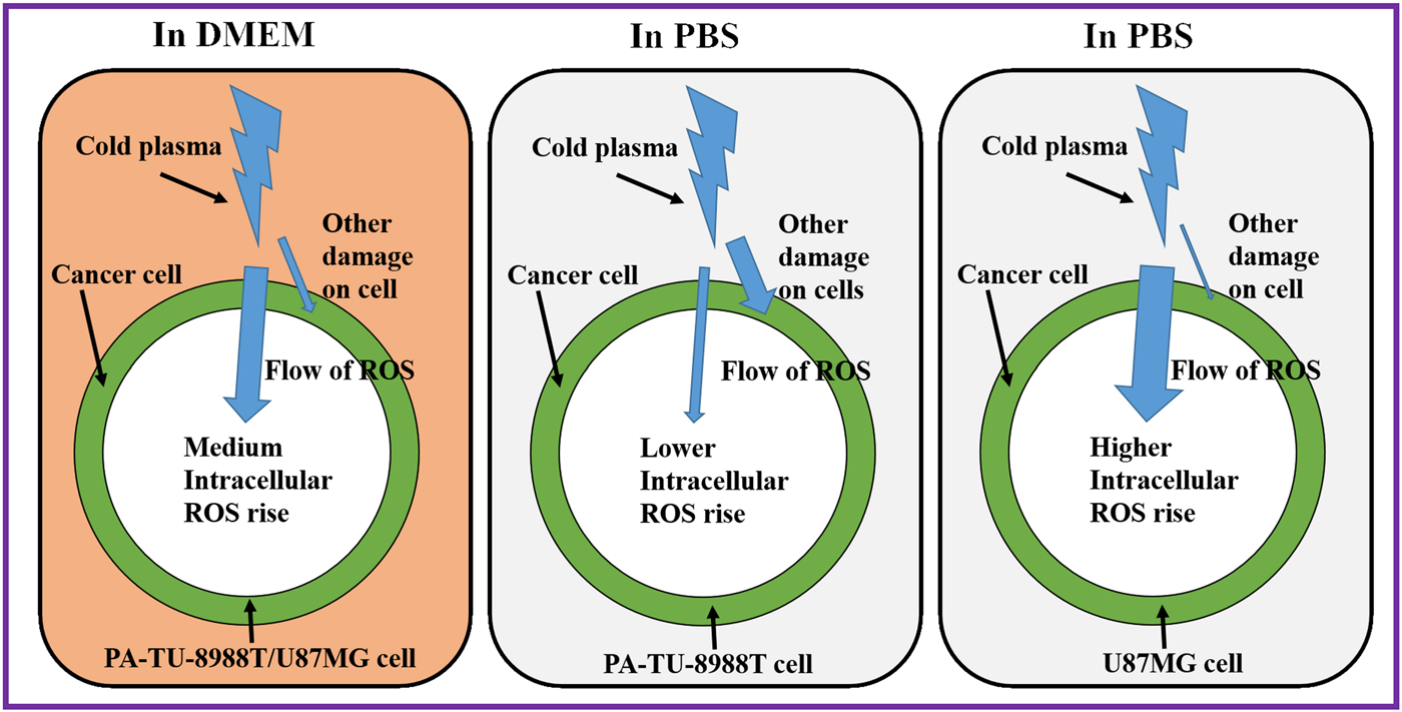
A schematic diagram to illustrate the proposed mechanism governing the different cellular responses in the CAP-stimulated DMEM and PBS. The intracellular ROS-based cell death pathways and other death pathways such as the cellular membrane damage-based cell death may coexist after the indirect CAP treatment.

Understanding the different intracellular ROS levels in pancreatic adenocarcinoma cells and glioblastoma cells affected by PSM and PSB may be the key to explain the distinct responses of cancer cells to PSM and PSB. We propose that different cellular responses in the nutrient-rich and the nutrient-starved environment may cause cancer cells to show different responses to the extracellular reactive species such as H_2_O_2_ (Fig. 8). When cells are cultured in a nutrient-rich environment such as in DMEM, most reactive species such as H_2_O_2_ may diffusion into the cytosol of cancer cells. The damage on the cellular membrane may be a minor factor to cause the cell death. Recently, we have demonstrated that the diffusion process is controlled by aquaporins; an important membrane channel for H_2_O_2_^62^. Knocking out one specific aquaporin, AQP8, significantly weakens the anti-cancer capacity of PSM on U87MG cells^62^. When cells are in PBS, the nutrient starvation may force cancer cells to enter an unusual state. For PA-TU-8988T cells, the nutrient starvation may inhibit the transmembrane diffusion of reactive species and inhibit the rise of intracellular ROS via an unknown mechanism. As a result, the damage on the cellular membrane may be stronger than that occurs in a nutrient-rich environment such as in DMEM. For U87MG cells, the nutrient starvation may enhance the transmembrane diffusion of reactive species and the rise of intracellular ROS also via an unknown mechanism. And, based on this model, it is possible to explain the observation in Fig. 3c and d that pre-treating cells with NAC can just counterpart the killing effect of PSB on U87MG cells rather than on PA-TU-8988T cells. Pre-treating cancer cells with NAC can just scavenge the intracellular ROS, rather than the extracellular ROS. In addition, the rise of intracellular ROS may also be due to other intracellular mechanisms, such as the generation of mitochondrial ROS in the early programmed cell death^63^. Further research on this mechanism governing the cell-dependent rise of ROS and other cellular damages such as membrane damage due to PSS is needed.

In our previous studies, several important factors which will affect the anti-cancer capacity of PSM have been proposed^21^. These factors include the contact area between CAP and the solution, the volume of the solution, as well as the distance between the CAP source and the solution^21^. A reasonable controlling these factors will obtain a stronger PSM without increasing the treatment time and dose. This study demonstrates a novel important factor, the dilutability of PSS. For some cell lines such as PA-TU-8988T and MDA-MB-231, the decrease of the nutrient components in DMEM will significantly weaken the anti-cancer capacity of PSM. For U87MG cells, a much weaker but similar trend has also observed as long as the dilution fraction is not too large; i.e. 90%. The impact of the dilution effect may be partially due to that fact that the cancer cells’ response to PSS is specifically determined by both the type of cancer cell and the components of solutions used to make PSS. In short, the biologically adaptable solutions such as PBS, DMEM, as well as the diluted DMEM are not just the carriers for the CAP-originated reactive species but also actively affect the killing process of PSS on cancer cells.

## Conclusions

In summary, the anti-cancer capacity of the cold plasma-stimulated solutions is both cell-dependent and dilutability-dependent. Pancreatic cancer cells and glioblastoma cells are highly vulnerable to the cold plasma-stimulated DMEM and the cold plasma-stimulated PBS, respectively. Such cell-dependent response may be due to the differential rise of intracellular reactive oxygen species of cancer cells in the specific H_2_O_2_-containing environment. However, the cold plasma-stimulated solutions alone can be a strong anti-cancer tool as long as the CAP treatment is of adequate duration long. Moreover, the dilutability of the solution is a very important factor affecting the anti-cancer capacity of the cold plasma-stimulated solution. For pancreatic cancer cells and breast cancer cells, the dilution on medium will significantly weaken the toxicity of the cold plasma-stimulated medium over these cells. For glioblastoma cells, the dilution will not noticeably affect the anti-cancer capacity of the cold plasma-stimulated medium.

## Electronic supplementary material

Fig. S1.
