# Supplementary material for: The Specific Vulnerabilities of Cancer Cells to the Cold Atmospheric Plasma-Stimulated Solutions
Source: Sci Rep. 2017 Jun 30;7:4479. doi: 10.1038/s41598-017-04770-x (PMC5493667; doi:10.1038/s41598-017-04770-x)
Supplement: Supplementary file 1 — Fig. S1. [file 41598_2017_4770_MOESM1_ESM.pdf]

The Specific Vulnerabilities of Cancer Cells to the Cold Atmospheric Plasma-Stimulated Solutions

Dayun Yan[1*], Haitao Cui[1], Wei Zhu[1], Niki Nourmohammadi[2], Julian Milberg[3], Lijie G. Zhang[1], Jonathan H. Sherman[4], Michael Keidar[1]*.

[1]Department of Mechanical and Aerospace Engineering, The George Washington University, Science & Engineering Hall, 800 22nd Street, NW, Room 3550, Washington, DC 20052, USA

[2]Department of Epidemiology, Columbia University, Mailman School of Public Health, 722 West 168th Street, New York, NY 10032, USA

[3]Department of Biomedical Engineering, University of Miami, 1251 Memorial Drive McArthur Engineering Building, Coral Gables, FL, 33146-0621, USA

[4]Neurological Surgery, The George Washington University, Foggy Bottom South Pavilion, 22nd Street, NW, 7th Floor, Washington, DC 20037, USA

*Corresponding authors: Dr. Dayun Yan: ydy2012@gwmail.gwu.edu,

Dr. Michael Keidar: keidar@gwu.edu.

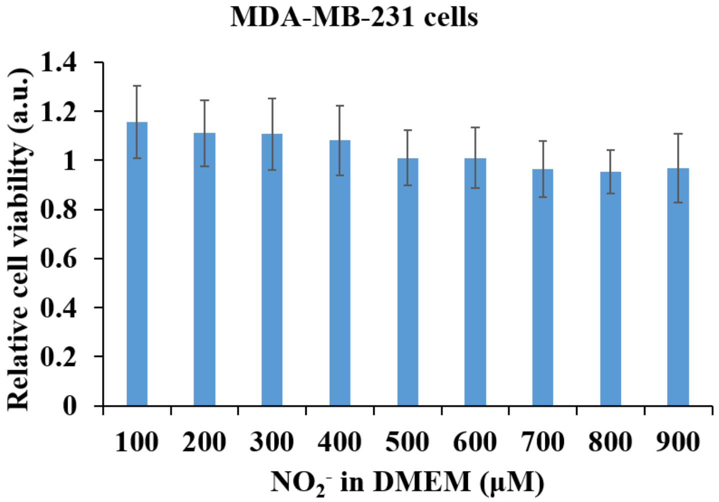

Fig. S1. The toxicity of NO_2^- on MDA-MB-231 cells. Results are presented as the mean ± s.d. of three independently repeated experiments performed in sextuplicate.
